# Comparison of volumetric responses to different corticosteroid administration methods in IgG4-related ophthalmic disease

**DOI:** 10.1371/journal.pone.0332392

**Published:** 2025-11-03

**Authors:** Min Kyu Yang, Seong Jung Ha, Ho-Seok Sa

**Affiliations:** Department of Ophthalmology, Asan Medical Center, University of Ulsan College of Medicine, Seoul, Republic of Korea; National Research Council (CNR), ITALY

## Abstract

**Purpose:**

To analyze the clinical and volumetric responses to different corticosteroid administration methods for IgG4-related ophthalmic disease (IgG4-ROD).

**Methods:**

The medical records of patients with bilateral lacrimal gland (LG) enlargement diagnosed with IgG4-ROD through unilateral LG biopsy between January 2011 and January 2022 were retrospectively reviewed. Clinical signs and the non-biopsied LG volume across three administration routes were compared: oral prednisolone (Pd), intravenous (IV) methylprednisolone (methylPd), and local triamcinolone (TA) injection. Radiological relapse was defined as the first instance of failure to satisfy the radiological response criteria, i.e., a post-treatment volume of <1.0 cm^3^ or a post-treatment to pre-treatment volume ratio of <35%.

**Results:**

Among the 28 patients, eight, ten, and ten received oral Pd, IV methylPd, and local TA injection, respectively. No significant differences were observed between the groups in terms of the baseline characteristics. Ophthalmic adverse effects were not observed in any patient. Kaplan–Meier survival analysis revealed that the 2-year radiological relapse-free survival in the IV methylPd group (80.0%) was higher than that in the oral Pd group (37.5%, p = 0.036) but comparable with that in the local TA group (100.0%, p = 0.886). The median post-treatment to pre-treatment volume ratio in the IV methylPd group (35.4%) was significantly lower than that in the oral Pd group (75.0%, p = 0.042) but comparable with that in the local TA injection group (38.5%, p = 0.321) one year after treatment.

**Conclusion:**

Compared with oral Pd, IV methylPd yielded better and more sustained radiological responses in patients with LG-involving IgG4-ROD. Local TA injection was also an effective alternative treatment option.

## Introduction

IgG4-related disease (IgG4-RD), an immune-mediated fibroinflammatory condition, is characterized by a dense lymphoplasmacytic infiltrate rich in IgG4-positive plasma cells and elevated serum IgG4 levels [1,2]. IgG4-related ophthalmic disease (IgG4-ROD) refers to the ocular adnexal or orbital manifestation of IgG4-RD. The lacrimal gland (LG) is the most frequently affected structure in patients with IgG4-ROD [3]. The affected LG may become enlarged and palpable, which manifests clinically as erythematous swelling of the upper lid or proptosis.

The oral administration of corticosteroids, which provide favorable responses in most cases, is the mainstay of the primary treatment for IgG4-ROD. Nevertheless, persistence of the lesions or relapse of IgG4-ROD has been commonly observed after oral steroid therapy [4,5]. Intravenous (IV) steroid pulse therapy significantly reduces the relapse rate compared with oral steroids, while maintaining the cumulative steroid dose and safety [6]. Andrew et al. [7] proposed that intraorbital triamcinolone (TA) injection may be a useful and safe alternative for the management of IgG4-ROD confined to the anterior orbit. However, repeated injections may be necessary owing to incomplete response and relapse.

The disease activity was assessed primarily based on qualitative changes in clinical signs in these studies of IgG4-ROD. Longitudinal measurements of quantitative indicators such as LG volume may facilitate the detailed assessment of disease activity, thereby enabling a more accurate comparison of the duration and degree of the response. Therefore, the present study analyzed the clinical and volumetric responses to various corticosteroid administration methods for IgG4-ROD.

## Methods

The medical records of patients with bilateral LG enlargement diagnosed with IgG4-ROD through unilateral LG biopsy between January 2011 and January 2022 were retrospectively reviewed. We designed the study in July 2021, and data were accessed for research purposes between December 2, 2021 and September 1, 2022. The patients were managed by two oculoplastic surgeons (H-SS and MKY) according to a common treatment approach at our institution (a 2700-bed academic tertiary referral hospital in Seoul, Korea). Patients who presented with swelling, redness, and palpable lesions of the eyelid or proptosis underwent contrast-enhanced orbital computed tomography (CT). Blood tests, including those for assessing autoimmune markers (e.g., serum IgG4), were conducted if CT revealed enhancing enlargement of the LG. Anterior orbitotomy and LG biopsy through the skin incision were performed on the larger of the two LGs. The clinical and histopathologic criteria established by the Japanese Study Group for IgG4-ROD were used to diagnose IgG4-ROD [8]. The exclusion criteria were as follows: the presence of concurrent extranodal marginal zone lymphoma, sclerosing histology, COVID-19 infection or vaccination during follow-up [9,10], post-treatment follow-up duration of <6 months, and the use of adjunctive treatments other than corticosteroid. The study adhered to the tenets of the Declaration of Helsinki. The study protocol was approved by the Institutional Review Board (approval no.: 2021−1323). Informed consent was waived since this research involves retrospective chart review and minimally harmful to participants. The data were analyzed anonymously. The individual pictured in Fig 3 has provided written informed consent (as outlined in PLOS consent form) to publish their image.

### Corticosteroid administration methods

Before treatment, systemic involvement was assessed by head/neck and truncal CT scans and positron emission tomography. Systemic corticosteroids administered orally or intravenously were considered the first-line treatment [6]. All patients treated between June 2012 and December 2016 received oral prednisolone (Pd) monotherapy [oral Pd group]. In brief, oral Pd was administered at a dose of 0.6 mg/kg/day for 4 weeks and tapered by 5 mg every 2–4 weeks. All patients treated between January 2017 and January 2022 received IV methylprednisolone (methylPd) alone [the IV methylPd group]. In brief, IV methylPd was administered once a week for 12 weeks (500 mg weekly for 6 weeks, then 250 mg weekly for 6 weeks). The subsequent dose of oral Pd was titrated based on the clinical response.

Localized TA injection (the local TA group) was primarily indicated for patients with localized involvement of LGs or systemic illnesses such as diabetes mellitus. In brief, 20 mg (0.5 mL, 40 mg/mL) of TA acetonide suspension (Shin Poong Co. Ltd., Seoul, Korea) was slowly injected into the palpated LG using a 27-gauge needle. The injection volume was reduced to 0.25 mL if significant resistance was encountered during the injection. Cold compression was applied for 6 h after the injection [11]. The patients were instructed to visit the emergency department if they experienced orbital pain, swelling, or sudden decline in vision. Low-dose oral Pd (5–10 mg/day) was administered to the participants in each treatment group if persistent inflammatory signs or abnormally high levels of serum IgG4 were detected.

### Clinical follow-up

The patients were monitored every 1–2 months for a minimum of 6 months, with follow-up intervals adjusted to 3–6 months based on the condition of the patient. The serum IgG4 levels were measured every 6 months, before and after the initial treatment and relapse. The complete resolution of erythematous eyelid swelling, proptosis, and LG palpability was defined as clinical response. A significant recurrence of any of these symptoms was defined as clinical relapse.

The systemic and ophthalmic adverse effects were assessed after completing corticosteroid treatment. The patients receiving TA injections underwent intraocular pressure (IOP) measurements, slit-lamp examinations, and fundus photography one week later to assess any acute complications [7]. Ophthalmic adverse effects included changes in the skin (depigmentation or TA deposition), ptosis, increased intraocular pressure (≥21 mmHg), retinal artery occlusion, and substantial cataract progression [7,12].

### Radiological follow-up

Follow-up CT scans were performed 3,6,12, and 24 months after completing corticosteroid treatment or when a clinical relapse was suspected. CT scans were not performed between the biopsy and the initiation of corticosteroid treatment; thus, LG volume analysis was performed only for the unoperated orbit to exclude the debulking effect of the biopsy. Volumetric analysis was performed using 3D modelling software (Mimics^®^ version 20.0, Materialise, Leuven, Belgium). A soft tissue window setting was used to differentiate the orbital fat from the extraocular muscle/LG [13]. A 3D LG model was created semi-automatically in the unoperated orbit by a single independent examiner (MKY). The mask threshold ranged from −30 to +150 Hounsfield units. Model volume calculation in the mask properties section was performed automatically using Mimics^®^. A post-treatment volume of <1.0 cm^3^ or a ratio of post-treatment volume to pre-treatment volume of <35% was defined as a radiological response. The first instance of failure to satisfy the criteria for radiological response (i.e., post-treatment volume of ≥1.0 cm^3^ and ratio of post-treatment volume to pre-treatment volume of ≥35%) was defined as a radiological relapse.

All statistical analyses were performed using the IBM SPSS Statistics software (version 21.0, IBM Corp., Armonk, NY, USA). The Mann–Whitney U test, Wilcoxon signed-rank test, and Kruskal–Wallis test were used to compare continuous variables. Fisher’s exact test was used to compare categorical variables. The Kaplan–Meier method was used to analyze the clinical and radiological relapse-free survival analysis. A two-sided p-value of <0.05 was considered statistically significant.

## Results

Among the 28 patients included in the present study (median age: 51.3 years; interquartile range [IQR]: 46.8–58.6), 16 (57.1%) were males. Eight, ten, and ten patients received oral Pd, IV methylPd, and local TA injection, respectively. No significant differences were observed among the three groups in terms of the baseline characteristics, including serologic features (Table 1). One patient in the local TA group received IV methylPd treatment 18.8 months before the TA injection, whereas four patients received oral Pd treatment at a median of 18.8 months prior (range, 1.8–28).

**Table 1:** Baseline characteristics of the patients with IgG4-related ophthalmic disease who received corticosteroids using various administration methods.

n = 28	Oral Pd (n = 8)	IV methylPd (n = 10)	Local TA (n = 10)	p value^a^
Age, years	50.6 (47.3–61.7)	53.4 (47.5–56.9)	51.4 (43.7–58.6)	0.817
Male sex, patients	5 (62.5)	5 (50.0)	6 (60.0)	0.851
History of corticosteroid treatment (within 3 years)				
Oral Pd	N/A	0 (0)	4 (40.0)	N/A
IV methylPd	0 (0)	N/A	1 (10.0)	N/A
Other ophthalmic manifestations				
Orbital nerve	0 (0)	2 (20.0)	1 (10.0)	0.407
Extraocular muscle	0 (0)	2 (20.0)	1 (10.0)	0.407
Systemic manifestation, patients				
Paranasal sinuses	5 (62.5)	6 (60.0)	8 (80.0)	0.407
Salivary glands	4 (50.0)	3 (30.0)	2 (20.0)	0.407
Lung	0 (0)	1 (10.0)	0 (0)	0.407
Pancreas	0 (0)	0 (0)	0 (0)	N/A
Kidney	1 (12.5)	1 (10.0)	1 (10.0)	0.603
Serologic features, patients				
Eosinophil >7%	3 (37.5)	2 (20.0)	0 (0)	0.125
IgG4 before treatment, g/L	1.87 (0.22–2.34)	4.92 (2.56–7.05)	4.39 (0.67–5.40)	0.071
IgG4 before treatment > 1.35 g/L	4 (50.0)	8 (80.0)	6 (60.0)	0.538

IV, intravenous; N/A, not applicable; Pd, prednisolone; TA, triamcinolone.

Data are presented as n (%) for categorical variables and median (interquartile range) for continuous variables.

a Comparison among the three groups using the Kruskal–Wallis test.

The median follow-up period following corticosteroid treatment was 19.4 months (IQR, 10.1–35.4) for all patients. The median follow-up period of the IV methylPd group (30.2 months, IQR 18.8–43.7) was longer than those of the oral Pd (14.7 months, IQR 9.9–34.9) and local TA (14.0 months, IQR 9.9–30.4) groups; however, the differences were not statistically significant (p = 0.091 and 0.096, respectively; Mann–Whitney U test). No ophthalmic or systemic adverse effects requiring treatment discontinuation (e.g., infectious disease, arrhythmia, cushingoid feature, acute hepatic failure) were observed in the three groups.

**Fig 1:**
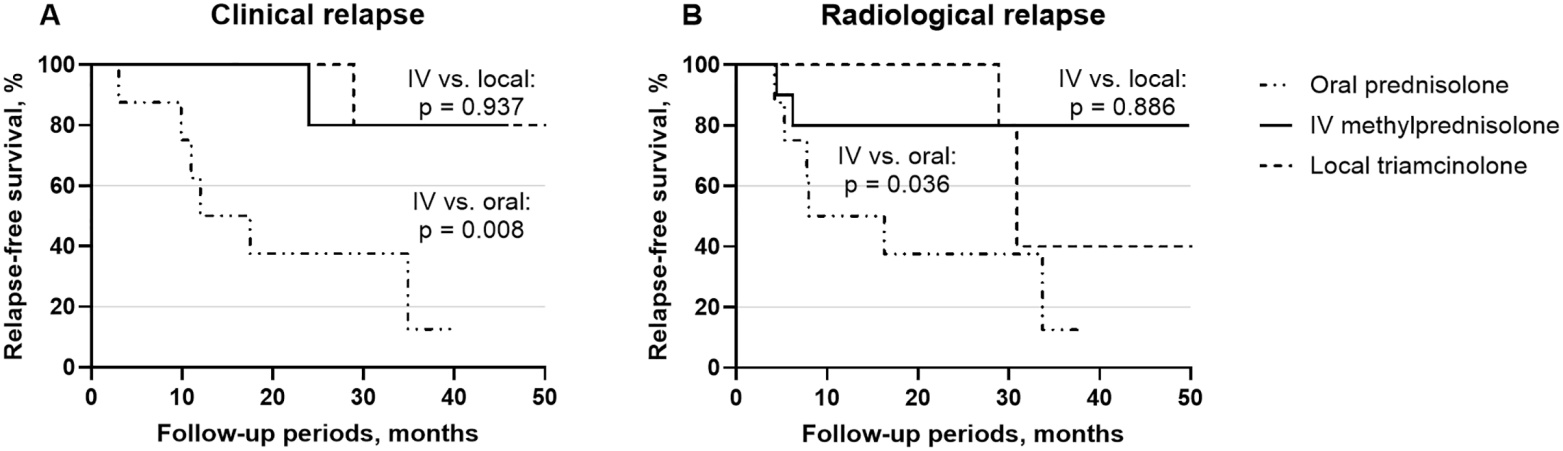
Kaplan–Meier analysis of (A) clinical and (B) radiological relapse-free survival for IgG4-related ophthalmic disease. Compared with oral prednisolone, intravenous methylprednisolone treatment resulted in a greater relapse-free survival. The relapse-free survival of the intravenous methylprednisolone group was similar to that of the local triamcinolone injection group.

Kaplan–Meier survival analysis revealed that the 2-year clinical relapse-free survival rate was 80.0% (95% confidence interval [CI] 44.2–100.0) in the IV methylPd group (Fig 1A). It was greater than that of the oral Pd group (37.5%, 95% CI 3.3–71.7) and similar to that of the local TA group (80.0%, 95% CI 44.2–100.0) (p = 0.008 and 0.937, respectively). The 2-year radiological relapse-free survival rates was 80.0% (95% CI 54.8–100.0) in the IV methylPd group (Fig 1B). It was greater than that of the oral Pd group (37.5%, 95% CI 3.3–71.7) and similar to that of the local TA group (100.0%) (p = 0.036 and 0.886, respectively).

Clinical relapses in the oral Pd and IV methylPd groups were preceded by radiological relapses, with median time intervals of approximately 3.0 months (IQR, 1.2–4.9) and 6.3 months (IQR, 4.9–12.1), respectively. Two patients in the local TA group exhibited clinical and radiological relapse at 28.9 and 51.5 months post-treatment, respectively. One patient exhibited only radiological relapse at 30.9 months.

**Fig 2:**
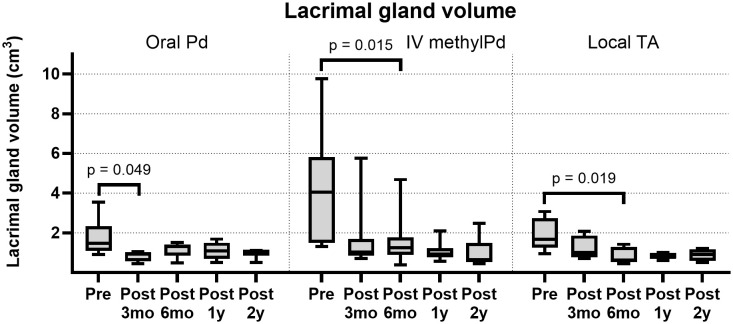
Longitudinal changes in the lacrimal gland (LG) volume of patients with IgG4-related ophthalmic disease. The intravenous methylprednisolone (IV methylPd) group and local triamcinolone (TA) group showed a gradual decrease in LG volume.

**Fig 3:**
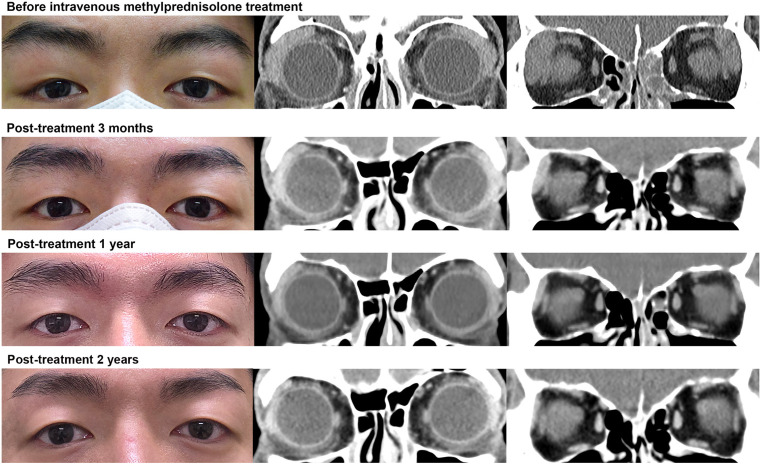
A representative case demonstrating the sustained lacrimal gland volume reduction effect of intravenous methylprednisolone treatment. Face photography and coronal computed tomography images (Top row) before treatment, (Second row) 3 months after treatment, (Third row) one year after treatment, and (Bottom row) 2 years after treatment. Gradual improvement in both upper lid lateral swellings was observed, along with a sustained decrease in lacrimal gland volume. The post-treatment to pre-treatment volume ratio of the left lacrimal gland was 38.4% at 3 months, 30.0% at 6 months, 21.6% at 1 year, and 18.7% at 2 years.

The LG volume exhibited a significant decrease at 3 months after oral Pd treatment (1.47 cm³ to 0.94 cm³, p = 0.049; Wilcoxon signed-rank test), which increased thereafter (Fig 2). In contrast, the LG volume in the IV methylPd and local TA groups exhibited a gradual decrease 2 years post-treatment, resulting in a significant difference when compared with the pre-treatment volume (p = 0.015 and 0.019, respectively, at 6 months post-treatment). The LG volumes were similar across the groups one year post-treatment (oral Pd: IV methylPd: local TA = 1.09 cm^3^: 0.93 cm^3^: 0.77 cm^3^, p = 0.283; Kruskal–Wallis test). Fig 3 shows a representative case demonstrating the sustained LG volume reduction effect of IV methylPd treatment. The serum immunoglobulin G4 level was stable throughout the follow-up period in this case (range: 3.75–4.06 g/L).

**Fig 4:**
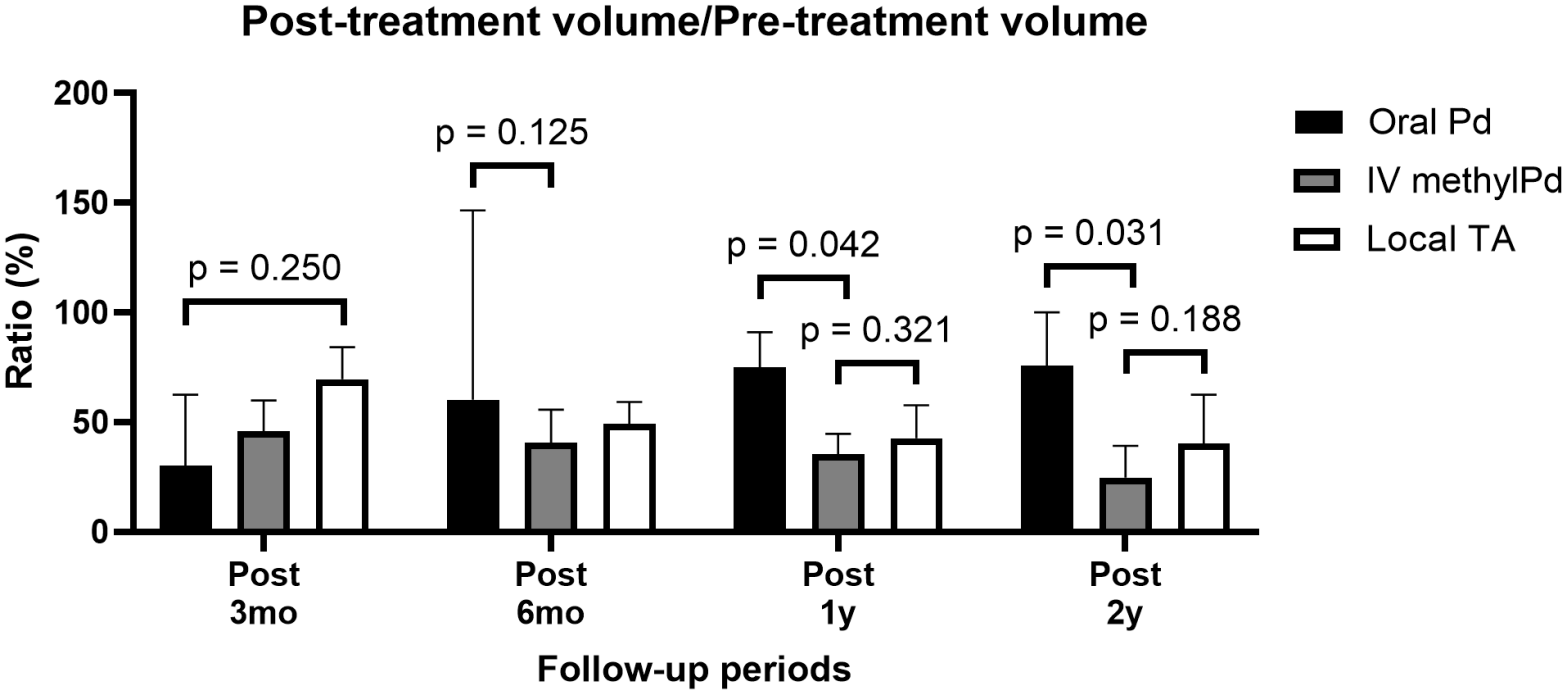
Longitudinal changes in the ratio of post-treatment volume to pre-treatment volume of the lacrimal gland. The median ratio of post-treatment volume to pre-treatment volume in the intravenous methylprednisolone (IV methylPd) group (35.4%) was significantly lower than that in the oral Pd group (75.0%, p = 0.042) and comparable with that in the local triamcinolone (TA) group (38.5%, p = 0.321) at one year post-treatment.

The pre-treatment LG volume in the IV methylPd group (4.05 cm^3^, IQR 1.52–5.61) was considerably larger than those in the oral Pd (1.47 cm^3^, IQR 1.18–2.32) and local TA (1.67 cm^3^, IQR 1.31–2.69) groups. Therefore, the ratio of post-treatment volume to pre-treatment volume was also calculated to compare the efficacy of LG volume reduction. The median ratio in the oral Pd group (35.6%) was similar to that in the IV methylPd group (45.9%, p = 0.527) 3 months post-treatment (Fig 4). The median ratio in the IV methylPd group (35.4%) was significantly lower than that in the oral Pd group (75.0%, p = 0.042) and comparable with that in the local TA group (38.5%, p = 0.321) at one year post-treatment.

## Discussion

The present study on IgG4-ROD with LG enlargement revealed that radiological relapse based on LG volume was closely associated with clinical relapse and typically preceded it. Comparison between various corticosteroid administration methods revealed that the rates of clinical and radiological relapse observed following IV methylPd treatment were comparable with those observed following local TA injection and superior to those observed with oral Pd treatment. Notably, in contrast to that observed with oral Pd treatment, the LG volume reduction effect of IV methylPd treatment persisted for over a year.

Precise assessment of disease activity aids in determining response and relapse, as well as evaluating treatment efficacy in the management of IgG4-ROD. The responder index has been validated to assess the activity of systemic IgG4-RD [14]. However, its sensitivity for IgG4-ROD localized in the orbit may be insufficient. A previous study on refractory orbital inflammation used the modified Werner’s classification score and the physician’s global assessment [15]. However, these have limited discriminability and reproducibility for inflammation localized in the anterior orbit [16].

Image analysis facilitates the objective assessment of the activity of IgG4-ROD. CT-based volumetric analysis of orbital soft tissue has been shown to be a reliable and feasible technique for the assessment of active thyroid eye disease [13]. CT scans of patients with IgG4-ROD typically reveal well-demarcated LG enlargement with homogeneous enhancement [17], thereby facilitating simple and reliable volume measurement [18].

Several previous studies have demonstrated the utility of ^18^F-fluorodeoxyglucose positron emission tomography/CT (^18^F-FDG PET/CT) and metabolic volume-based analysis in the evaluation of IgG4-RD activity [19,20]. However, its lower accessibility limits the application of ^18^F-FDG PET/CT for serial post-treatment follow-up examinations. MRI is also less accessible than CT due to its significantly higher cost and longer appointment wait times. Therefore, CT is a more practical option for follow-up monitoring in clinical settings.

In terms of safety, the radiation dose from a single head CT scan is approximately half of the annual natural background radiation [21,22]. While serial diagnostic head CT may slightly increase the risk of cataract formation [22], the associated risk of radiation-induced cancer is generally considered negligible, especially in elderly individuals [23,24].

Given its reliability, accessibility, and acceptable safety profile, CT-based measurement of LG volume can be used as an objective and quantitative indicator for evaluating activity in IgG4-ROD, as the LG is the most frequently affected organ in patients with IgG4-ROD and its size decreases in response to treatment [25]. Furthermore, the involvement of multiple LGs and/or major salivary glands is associated with greater systemic disease activity [26,27].

No previous study has evaluated treatment response by measuring volume, even in systemic IgG4-RD [28]. A study involving patients with IgG4-RD in China [29] revealed that the affected LG volume was 2.103 ± 0.714 cm^3^ and 2.256 ± 1.236 cm^3^ in middle-aged male and female patients, respectively. The normal LG volume varied from 0.606 ± 0.048 cm^3^ to 1.55 ± 0.40 cm^3^ in middle-aged participants in several Korean studies [11,30,31]. Thus, a post-treatment LG volume of <1.0 cm³ was deemed indicative of a response. The radiological response evaluation criteria in lymphoma (RECIL 2017) was adopted in the present study [32], as the characteristics of lymphoma have similarities to those of IgG4-ROD in terms of LG involvement, steroid responsiveness, and histologic features [33]. A post-treatment volume to pre-treatment volume ratio of <35% was additionally included as a criterion for treatment response, given that a decrease in diameter of ≥ 30% in RECIL 2017 corresponds to a volume of <34.3%. The application of these criteria resulted in radiological relapse preceding each clinical relapse by 0–6 months.

The pre-treatment LG volume and serum IgG4 levels in the IV methylPd group were higher than those in the oral Pd group. Given these pre-treatment characteristics, a higher risk of relapse was expected in the IV methylPd group [18]. However, the actual radiological relapse-free survival was significantly greater in the IV methylPd group. Therefore, these characteristics are unlikely to affect the overall direction of the findings, making a stratified subgroup analysis based on pre-treatment LG volume or elevated serum IgG4 levels unnecessary.

The volume reduction effect in terms of the LG volume ratio was similar in oral Pd and IV methylPd groups at 3 months post-treatment (median: 35.6% vs. 45.9%). However, this effect declined after 3 months in the oral Pd group, whereas it persisted for >1 year post-treatment in the IV methylPd and local TA groups. Our previous study suggested that the better outcomes of IV methylPd treatment may be associated with a better initial response or the effect of the pulse regimen on circulating inflammatory cells [6]. The findings of the present study support the effect of the pulse regimen on circulating inflammatory cells as the etiology of long-term suppression of relapse. IV administration of high-dose steroids may achieve long-term suppression of relapse by completely abolishing circulatory dendritic cells, resulting in a more prominent decrease in T3/T4 lymphocytes or induction of epigenetic change [34–36].

The present study demonstrated the favorable long-term outcomes of a single local TA injection, which were superior to those of oral Pd treatment and comparable with those of IV methylPd treatment. Andrew et al. [7] reported that a single TA injection resulted in long-term clinical response in three out of 10 cases. Our radiological analysis provided objective evidence for the efficacy of local TA injection and comparisons with other treatment strategies. Local TA injection has the advantage of avoiding systemic side effects and being more convenient for patients. However, the accurate injection of the steroid into the LG can be technically challenging and is ineffective for systemic IgG4-RD. Thus, it can be considered a first-line treatment for IgG4-ROD localized in the anterior orbit or IgG4-ROD with systemic illness contraindicating the use of systemic corticosteroids. Further well-structured controlled trials must be conducted to determine whether TA injection could serve as a viable alternative to systemic steroids in such scenarios.

Our study has some limitations. First, half of the patients in the local TA injection group had received systemic corticosteroids previously. The intervals between TA injection and prior treatments were >19 months and >2 months following IV methylPd and oral Pd treatments, respectively. Therefore, the possibility of long-term effects from prior systemic corticosteroids cannot be excluded. Second, additional treatments owing to clinical relapse resulted in the exclusion of subsequent volume data from analysis. This censoring would likely lead to an underestimation of post-treatment volume, especially in the oral Pd group. Fortunately, this underestimation is not expected to alter the overall direction of our findings.

In conclusion, longitudinal LG volume measurement may facilitate a more sensitive and objective assessment of treatment response. IV methylPd yields a better and more sustained radiological response than oral Pd for LG-involving IgG4-ROD. Local TA injection is another effective alternative treatment option.
